# Vertebrobasilar artery elongation in migraine—a retrospective cross-sectional study

**DOI:** 10.1007/s13760-022-02039-3

**Published:** 2022-07-29

**Authors:** Ole Hensel, Philipp Burow, Torsten Kraya, Dietrich Stoevesandt, Steffen Naegel

**Affiliations:** 1 Department of Neurology, Martin-Luther-University Halle-Wittenberg, University Hospital Halle, Halle (Saale), Germany; 2 Department of Neurology, St. Georg Hospital Leipzig, Leipzig, Germany; 3 Department of Radiology, Martin-Luther-University Halle-Wittenberg, University Hospital Halle, Halle (Saale), Germany

**Keywords:** Migraine, Vertebrobasilar dilation and elongation, Outlet angle of the superior cerebellar artery, Lateral displacement of the basilar artery

## Abstract

**Background:**

Numerous but inconclusive findings have sparked an ongoing debate about whether the arteries of migraine patients undergo vascular alterations. The outlet angle of the superior cerebellar artery (SUCA) and the lateral displacement of basilar arteries are good surrogate parameters for determining elongation of the vertebrobasilar arteries.

**Methods:**

We retrospectively determined the SUCA outlet angle and the lateral displacement of the basilar artery in 63 patients with migraine (30.6 ± 8.9 years, 84% women, 16% chronic migraine, 60% migraine with aura) and compared these with 126 age- and sex-matched control subjects.

**Results:**

In patients with migraine, the SUCA outlet angle was lower (159 ± 26° vs. 169 ± 29°, *p* = 0.020) and the lateral displacement of the basilar artery was greater (3.7 ± 2.7 mm vs. 2.8 ± 2.4 mm, *p* = 0.020) than in the control subjects. Age, gender, migraine characteristics and presence of any cardiovascular risk factors did not affect the SUCA outlet angle or lateral displacement of the basilar artery.

**Conclusion:**

Migraine patients exhibited a lower SUCA outlet angle and greater lateral displacement of the basilar arteries. Both may be attributable to the elongation of the vertebrobasilar arteries, which is an indication of arterial wall pathology in migraine.

## Introduction

Vascular alterations in migraine patients have long been suspected, and their role in migraine disease has been the subject of repeated discussions [1]. Not only are vascular alterations discussed in the context of the pathophysiology of migraines, but there are also indications that the migraine itself may lead to vascular alterations. Recently, meta-analyses of genome-wide studies detected over 100 gene loci associated with migraine. Few of these genes (e.g., LRP1) are associated with vascular function [2, 3]*.* LRP1, for instance, is associated with the integrity of the arterial vessel wall [4] and is the gene most highly correlated with migraine disease [2, 3]. The aorta, other large arteries, and internal jugular veins have been shown to be stiffer in migraine patients than in healthy controls [5–8]. Meta-analyses have revealed an increased pulsatility index and decreased cerebrovascular responsiveness to hypercapnia in the posterior circulation of migraine patients [9]. Patients with migraine are also more likely to have an abdominal aortic aneurysm (adjusted HR 3.558, 95% CI = 1.439–8.799) [10] or coronary artery dissection (incidence ratio 1.37, 95% CI 1.05–1.76) [11]. Migraineurs also have a slightly but significantly increased risk for developing spontaneous cervical artery dissections (OR 2.06, 95% CI 1.33–3.19) [12], white matter lesions (multiple adjusted OR 1.6, 95% CI 1.0–2.7; OR 3.9, 95% CI 2.26–6.72) [13, 14], hemorrhagic strokes (adjusted effect estimate 1.48, 95% CI 1.15–1.90; crude HR 2.22, 95% CI 1.78–2.77; adjusted HR 1.41, 95% CI 1.25–1.61) [15–17], and infratentorial cerebral microbleeds (OD 6.1, 95% CI 1.5–25) [18]. Patients with migraine also have an increased risk of trigeminal neuralgia (HR 6.72, 95% CI 5.37–8.41) [19] and are more prone to developing subsequent ocular motor cranial nerve palsies (HR, e.g., fourth cranial nerve 4.23, 95% CI 2.04–8.76) [20]. Furthermore, migrainous infarctions are predominantly located in the vertebrobasilar circulation [21, 22]. Patients with aura tend to have greater lateral displacement of the basilar artery (BA, 6.3 ± 3.8 mm vs. 4.9 ± 3.1 mm; *p* = 0.055) [23]. A recent study showed that the greater BA curvature was associated with patients with aura (OR 1.042 per mm, 95% CI 1.006–1.080) [24].

These vascular findings and complications suggest arterial wall weakness of the vertebrobasilar artery as well as other arteries, potentially indicating an arterial wall pathology. A weakness of the vessel walls leads to both dilation and elongation; the weakening of circular wall structures leads to dilation, while the weakening of longitudinal wall structures leads to elongation. Dilation and elongation are both characteristics of dolichoectasia [25], which often involves the basilar artery [26]. Elongation is easier to detect than dilation because the effects accumulate over a longer distance. Such elongation of the basilar artery may result in lateral displacement of its course, or a cranial shift in the distal basilar artery. Measurement of the superior cerebellar artery (SUCA) outlet angle has recently been proposed as a surrogate parameter for the cranial shift of the distal basilar artery [27].

In this study, we investigated whether patients with migraine have an elongation of the basilar artery compared to sex- and age-matched control subjects. This could indicate a relationship between vertebrobasilar elongation and the occurrence of migraine.

## Methods

### Patients and control subjects

**Fig. 1:**
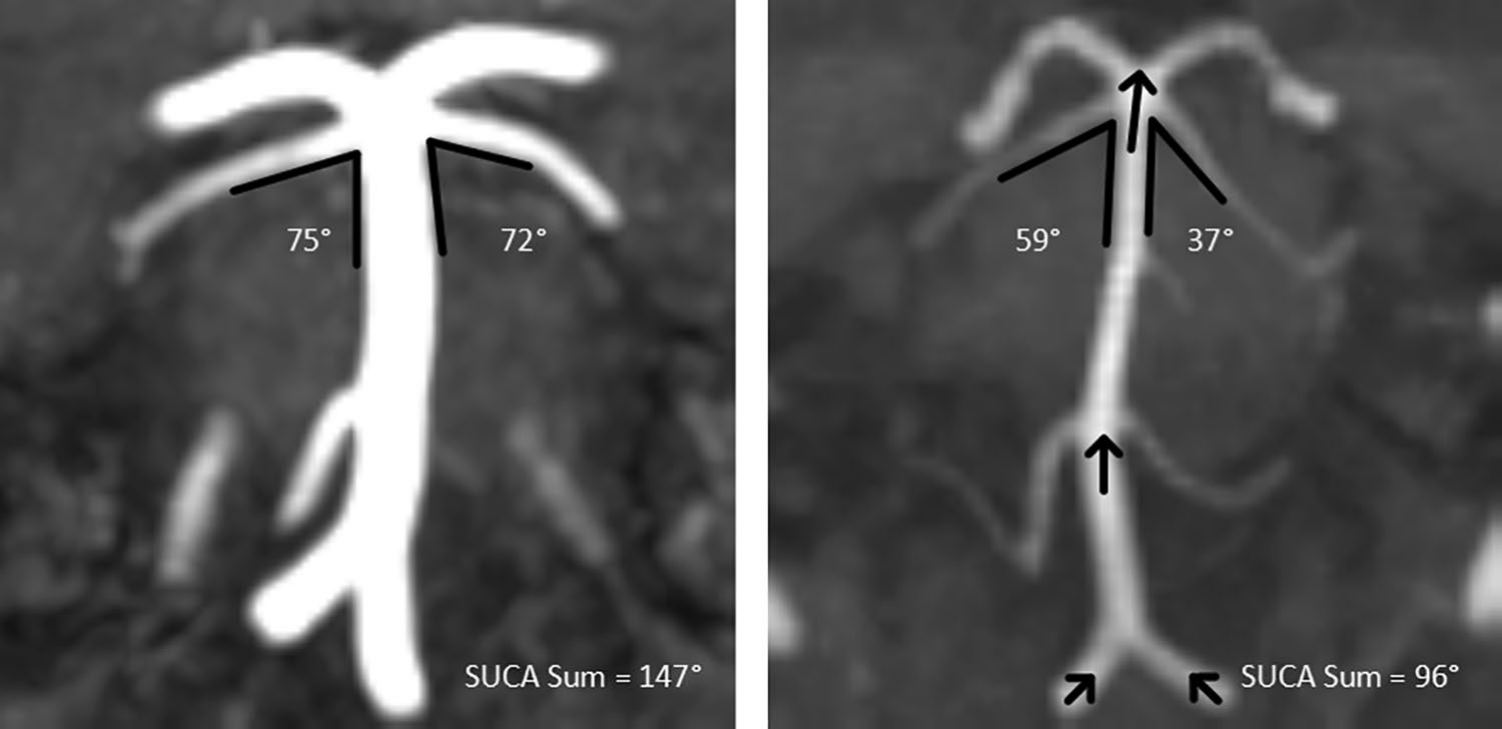
Coronal TOF-MRAs measuring the SUCA outlet angles of two patients with migraine (SUCA outlet angle in left image: 147°, in right image: 96°). The arrows show elongation and cranial displacement of the vertebrobasilar arteries. Note: The distal vertebral artery is attached to the dura mater as it passes from an extracranial to an intracranial region (not shown).

The retrospective study was approved by the ethics committee of the Faculty of Medicine at Martin-Luther-University Halle-Wittenberg (reference number: 2021–058). A waiver of informed consent was granted by the ethics committee for all participants. The study was performed in accordance with relevant guidelines and regulations. The study began by assessing all patients who were hospitalized in the Department of Neurology at University Hospital Halle between 2/2001 and 2/2021 and who received the coded discharge diagnosis of migraine (ICD-10: G 43.x). Migraine was either the main or concomitant diagnosis. Next, all patients with no MRI or an unsuitable MRI (e.g., lack of coronal TOF-MRA imaging) were excluded. Afterwards, migraine diagnoses were further reviewed retrospectively using discharge letters or other clinical documentation. Headaches were classified based on the International Classification of Headache Disorders—ICHD-3 [28] by a neurologist (PB) from a tertiary headache center who specializes in headaches. Thus, only patients for whom the individual criteria for migraine were testable according to ICHD-3, e.g., from detailed medical histories, were included in the migraine group. Patients were excluded if the detailed history revealed evidence of another primary or secondary headache disorder, or the information was not sufficient enough to classify the headache. The same applied to control subjects. Figure 1 shows a flowchart of how patients were selected for the analysis.

Patients and controls were included only if the branches of both SUCA from the basilar artery onwards and the entire course of the basilar artery could be reliably visualized on coronal TOF-MRA imaging (more details below). TOF-MRA imaging was performed with two scanners (3.0°T Skyra and 1.5 T Sonata Vision, both Siemens, Erlangen, Germany).

Controls subjects (*n* = 126, age 30.4 ± 8.9 years, 106 women) were matched (1:2) by age (± 2 years) and sex. MRI imaging had been clinically performed in the past for one of the following medical reasons: multiple sclerosis, optic neuritis, non-macroangiopathic TIA (or minor stroke), first-time epileptic seizure, or vertigo.

To prevent bias, migraine patients and controls with observed macrovascular alterations (e.g., carotid stenosis > 50%) were excluded. Patients suffering from diseases known to be associated with vascular alterations, such as dissection, aneurysm, arteriovenous malformation, vasculitides, veins, connective tissue disorders (e.g., fibromuscular dysplasia, Marfan syndrome, Ehler-Danlos syndrome, Loeys-Dietz syndrome), kidney disease, active tumor diseases, lysosomal storage diseases, or anemia were also excluded. Pathological processes near the basilar head (e.g., abscess, pituitary adenoma, or meningioma in the region of the clivus) also led to the exclusion of patients.

Data were collected on age, sex, cardiovascular risk factors (arterial hypertension, diabetes mellitus, hyperlipidemia, smoking, obesity), presence of aura, and frequency of migraine attacks (< or ≥ 15 days of headache per month).

The SUCA outlet angle and the lateral displacement of basilar arteries were measured on an anonymous basis (OH) in TOF-MRA images with a maximum intensity projection (MIP). The data are reported as mean ± SD.

### SUCA outlet angle

The SUCA arises from the distal end of the basilar artery and has comparatively little variation [29]. The SUCA passes under the third cranial nerve and takes the shortest route to the tentorium cerebelli in order to supply the superior parts of the cerebellum. At a fetal age, the SUCA outlet angle is greater than 170° [30, 31] and decreases with age [27]. The SUCA outlet angle is measured on coronal angiographic images *(*e.g., TOF-MRA, Gd-MRA or CTA) and according to the Smoker criteria [24] the SUCA outlet angle most likely corresponds to the height of the basilar head.

**Fig. 2:**
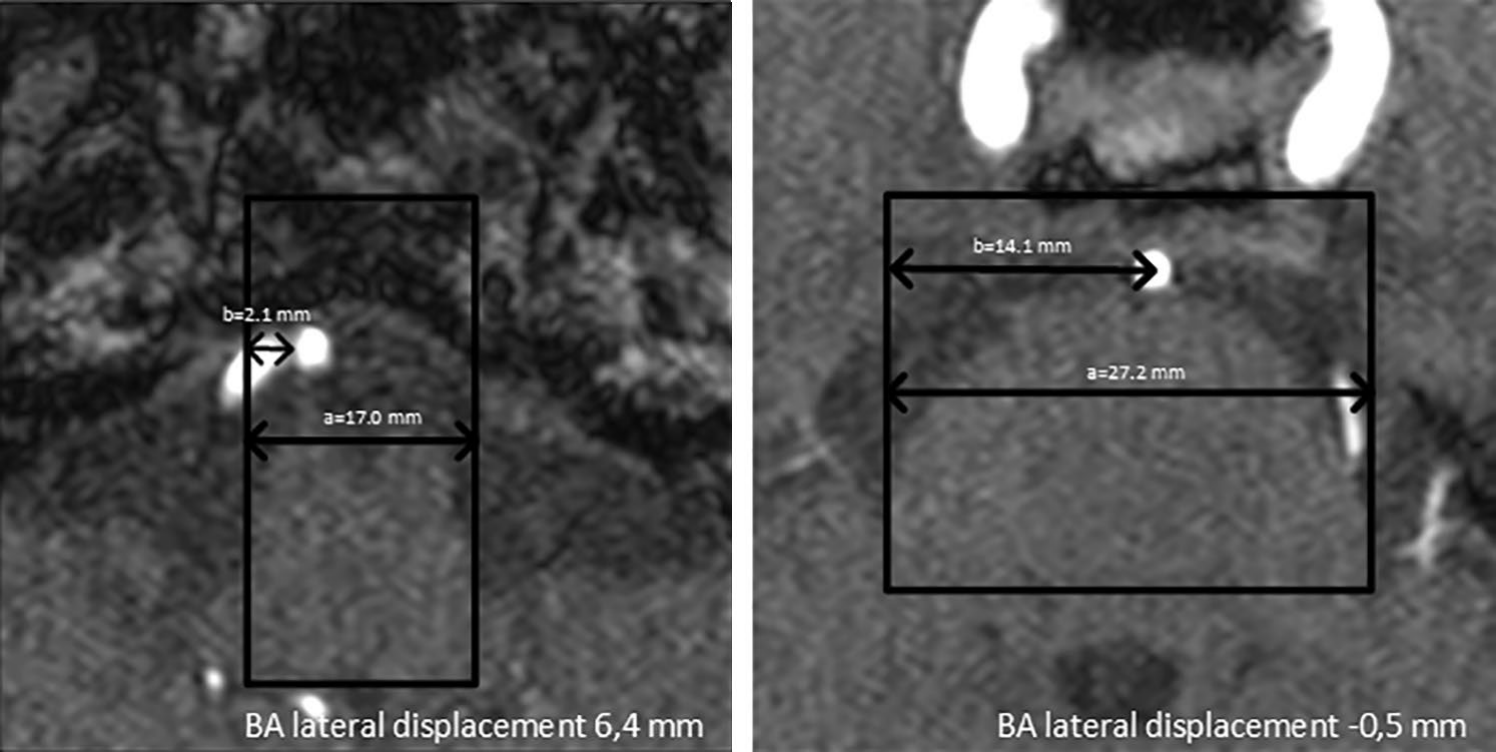
Axial TOF-MRA with measurement of the lateral displacement of the basilar artery (BA). A rectangle with the width of the brainstem (**a**) is created and the distance between the center of the artery center and the left side (**b**) is measured. The lateral displacement of the basilar artery is calculated using the formula (a/2)–b. The absolute values are used in the analysis

The proximal branch was used to measure the angle when two SUCA arose from the basilar artery on one side, or when the SUCA divided early. Patients or controls were excluded if bilateral SUCA outlets could not be visualized with certainty or if at least one SUCA originated from the head of the basilar artery or precommunicating segment of the posterior cerebral artery. The legs of the angle were applied to the SUCA or basilar artery 5 mm from the SUCA outlet (Fig. 2). The left and right SUCA outlet angles were added together.

### Lateral displacement of the basilar artery

**Fig. 3:**
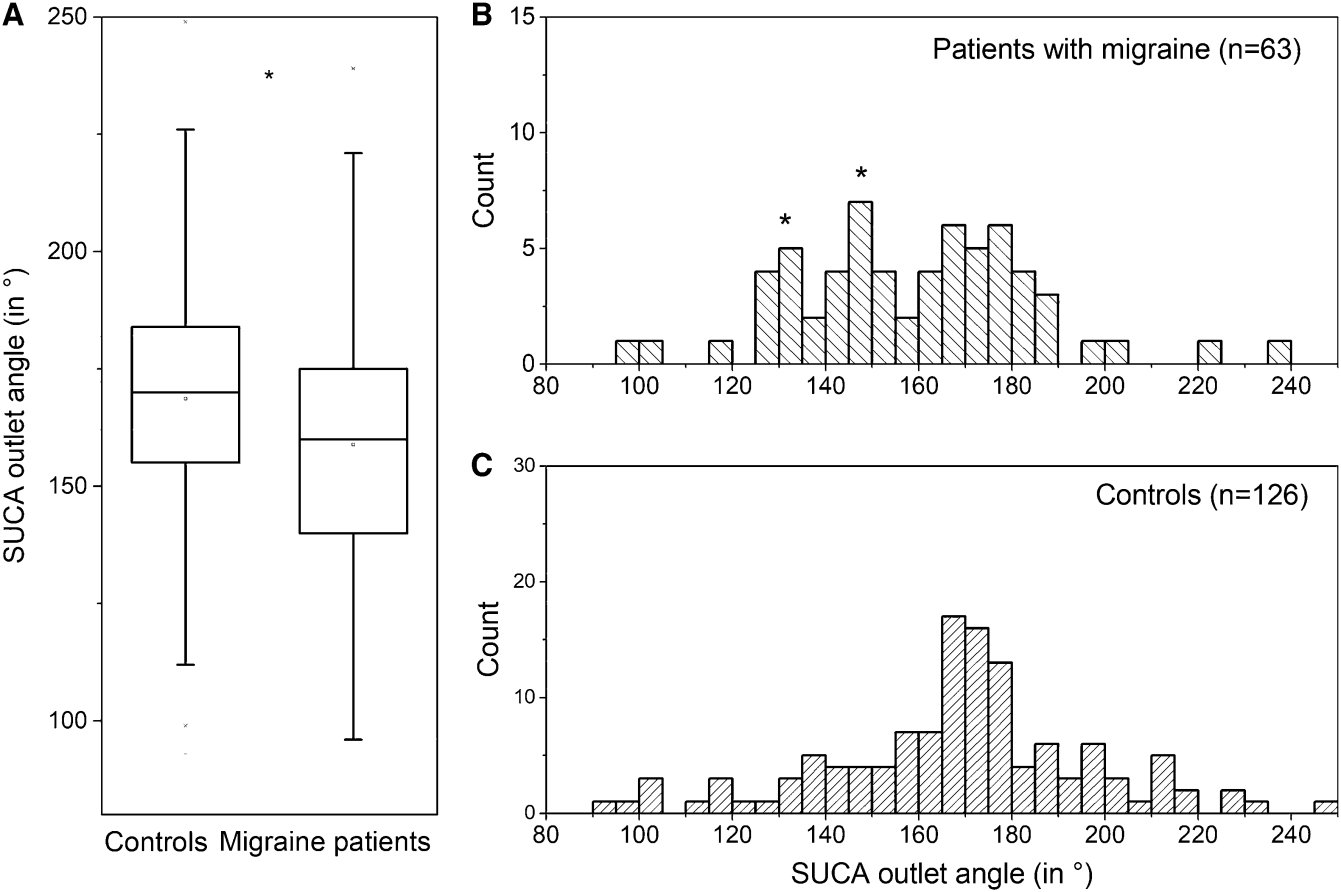
**A** SUCA outlet angles in controls and migraine patients (median line, range with 1.5IQR, 25–75%, * two sample *t*-test *p* = 0.020). **B** and **C** Frequency distribution of SUCA outlet angles in patients with migraine (**B**) and controls (**C**, each bar = 5°). Note the different y-scaling of **B** and **C** (1:2 matching). The frequencies of the migraine patients have a normal distribution and exhibit two additional peaks (* in **B**). The frequencies of the control group do not have a normal distribution

The lateral displacement of the basilar artery was measured in axial TOF-MRA images. The maximum lateral displacement of the basilar artery on axial imaging was identified and the distance was then measured between the center of the artery and the center of the brainstem (Fig. 3). The confluence was used if the maximum lateral displacement was equal to the beginning of the basilar artery. Absolute values in mm were used in the analysis.

## Results

Sixty-three patients could be included in the migraine group (age 30.6 ± 8.9 years; 53/63 women) and 126 patients (age 30.3 ± 8.9, 106/126 women) in the control group. Based on the ICHD-3, 81% (51/63) were classified as having episodic migraine and 16% (10/63) as having chronic migraine (information was insufficient for classification in the case of two patients). Sixty percent (38/63) of patients had a migraine with aura and 37% (23/63) patients had a migraine without aura (information was insufficient for classification in two patients). Among patients with aura, 53% (20/38) had visual or brainstem aura, while 45% (17/38) had other aura symptoms (information was insufficient for classification in one patient).

Cardiovascular risk factors were present in 11% (7/63) of the migraine patients. Coronary artery disease was not reported in any of the study participants. Controls generally had a higher rate of hyperlipidemia (chi-square test *p* = 0.057) and diabetes mellitus (chi-square test *p* = 0.078, Table 1).

**Table 1:** Cardiovascular risk factors in migraine patients and controls

	Migraine (*n* = 63)	Controls (*n* = 126)	Chi-square
Hypertension	3 (5%)	11 (9%)	0.32611
Diabetes mellitus	0 (0%)	6 (5%)	0.07837
Hyperlipidemia	0 (0%)	7 (6%)	0.05659
Smoking	4 (6%)	13 (10%)	0.36732
Obesity	4 (6%)	6 (4%)	0.64585

### SUCA outlet angle

**Fig. 4:**
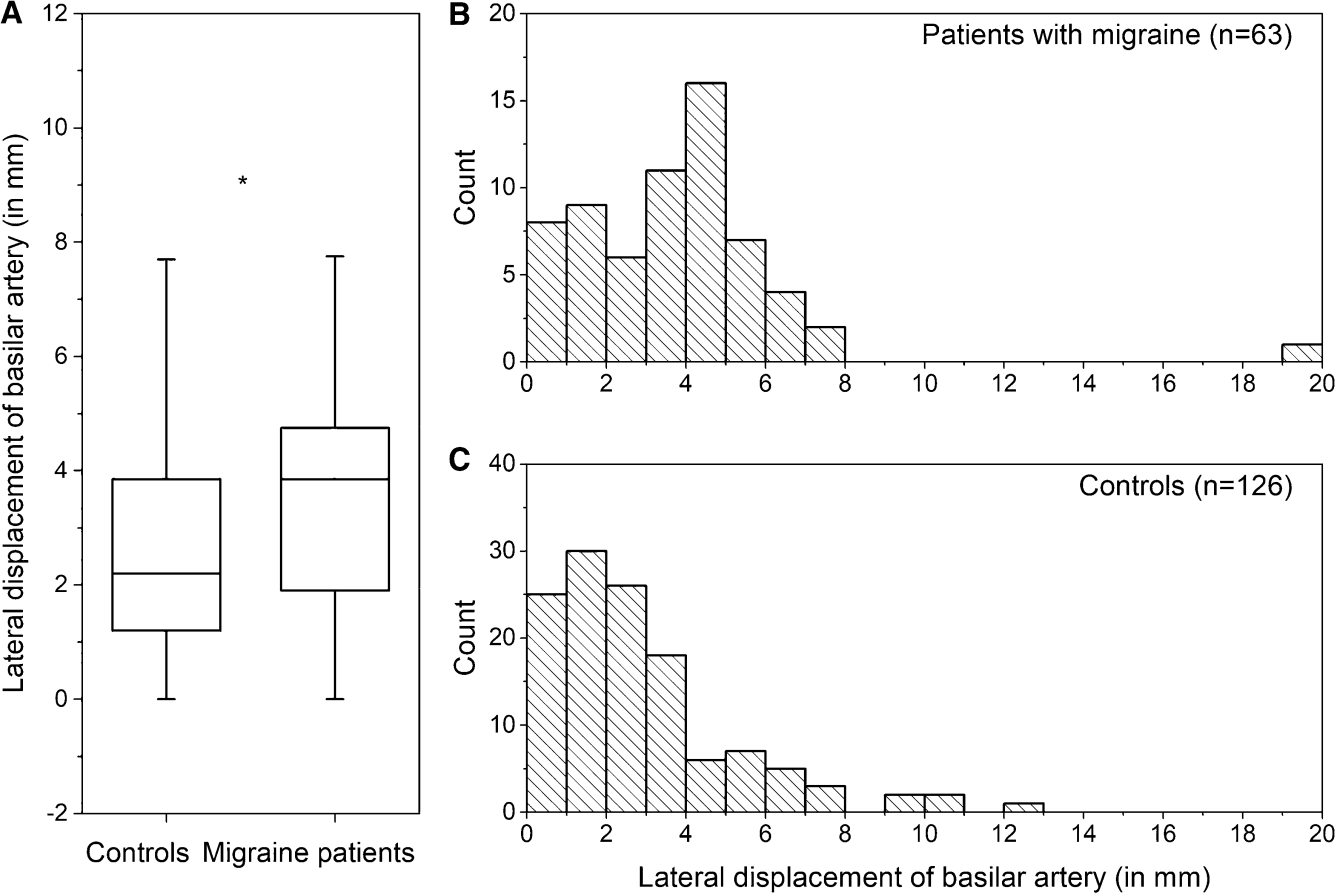
**A** shows a box plot of lateral displacement of the basilar artery in controls and migraine patients (median line, range with 1.5IQR, 25–75%, * two sample *t*-test *p* = 0.020). **B** and **C** Frequency distribution of lateral displacement of the basilar artery in patients with migraine (**B**) and controls (**C**, each bar = 1 mm). Note the different y-scaling of **B** and **C** (1:2 matching)

The outlet angle of the SUCA was lower in migraine patients than in the control subjects (159 ± 26° vs. 169 ± 29°, two sample *t*-test *p* = 0.020; Fig. 4A). There was no difference in the SUCA outlet angle of migraine patients with episodic and chronic courses (160.6 ± 25.1° vs.148.5 ± 29.9°, two sample *t*-test *p* = 0.184) nor between migraine patients with or without aura (155.6 ± 27.5° vs. 162.7 ± 23.0°, two sample *t*-test *p* = 0.303).

In migraine patients with aura, the SUCA outlet angle did differ from controls (156.7 ± 27.2° vs. 170.0 ± 30.6°, two sample *t*-test *p* = 0.0215), but not in migraine patients without aura (162.7 ± 23.0° vs. 166.3 ± 27.0°, two sample *t*-test *p* = 0.583). In patients with visual or brainstem aura, the SUCA outlet angle did not differ from patients with other aura symptoms (151.6 ± 32.6° vs. 161.6 ± 19.8°, two sample *t*-test *p* = 0.276).

Across all patients and controls (*n* = 189), the SUCA outlet angle was not affected by the presence of any of the cardiovascular risk factors (two sample *t*-test all *p* > 0.200). The angle did not differ between gender (women: 166 ± 30° vs. men 165 ± 23°, two sample *t*-test *p* = 0.951), and had no correlation with age (Pearson *r* = –0.12269, *p* = 0.093).

In migraine patients with aura, the lateral displacement of the basilar artery did differ from controls (4.2 ± 3.1 mm vs. 2.7 ± 2.1 mm, two sample t-test *p* = 0.003), but not in migraine patients without aura (3.0 ± 1.6 mm vs. 3.0 ± 2.9 mm, two sample *t*-test *p* = 0.907). In patients with visual or brainstem aura, the lateral displacement of the basilar artery did not differ from patients with other aura symptoms (4.1 ± 4.0 mm vs. 3.7 ± 1.9, two sample *t*-test *p* = 0.333).

In a multiple linear regression analysis, which included the factors age, gender, arterial hypertension, diabetes mellitus, smoking, hyperlipidemia, obesity, migraine and the dependent variable SUCA outlet angle, only the presence of migraine was significantly associated with an altered SUCA outlet angle (*p* = 0.031).

The frequency distribution of SUCA outlet angles showed multiple peaks in migraine patients, whereas only one peak was observed in the control subjects (Fig. 4B and 4C). Therefore, we conducted subgroup analyses as an exploratory approach. Taking the median SUCA outlet angle in migraine patients (160°) as a cut-off enabled subgroups with higher (*n* = 30, 48%) and lower (*n* = 33, 52%) SUCA angles to be identified. When these groups were compared, no difference in age, gender or risk factors were observed.

When this cutoff value was applied, 39 (31%) control subjects also had lower SUCA outlet angles, which is significantly less than in the migraine subgroup (chi-square test *p* = 0.004).

### Lateral displacement of the basilar artery

**Fig. 5:**
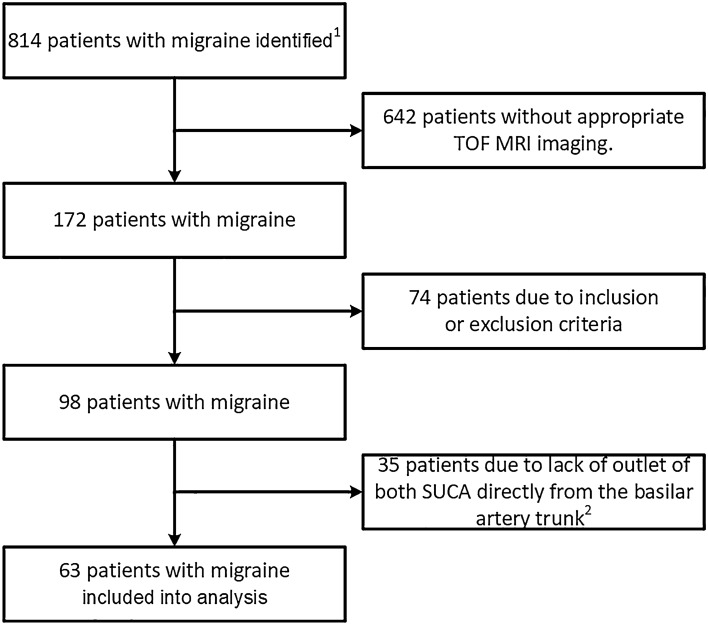
Flowchart of patient exclusion. **1** 814 inpatients were discharged with a diagnosis of migraine between 2/2001 and 2/2021. **2** To guarantee homogeneous measurements, patients in whom the superior cerebellar artery (SUCA) arises from the precommunicating segment of the posterior cerebral artery or directly from the widened head of the basilar artery were excluded

The basilar artery was located more laterally in patients with migraine than in the control subjects (3.7 ± 2.7 mm vs. 2.8 ± 2.4 mm, two sample *t*-test *p* = 0.020, Fig. 5A). The SUCA outlet angle decreased with the increasing lateral displacement of the basilar artery (Pearson *r* = –0.281, *p* = 0.000).

There was no difference in the lateral displacement of the basilar artery of migraine patients with episodic and chronic courses (3.6 ± 1.9 mm vs. 4.6 ± 5.3 mm, two sample *t*-test *p* = 0.267) nor between migraine patients with or without aura (4.2 ± 3.2 mm vs. 3.0 ± 1.6 mm, two sample *t*-test *p* = 0.099).

Across all patients and controls, the presence of any cardiovascular risk factor did not affect the lateral displacement of the basilar artery (two sample *t*-test all *p* > 0.200). The displacement did not differ between sexes (women: 3.0 ± 2.4 mm vs. men 3.7 ± 3.1 mm, two sample *t*-test *p* = 0.181), and had no correlation with age (Pearson *r* = –0.063, *p* = 0.627).

In a multiple linear regression analysis, which included the factors age, gender, arterial hypertension, diabetes mellitus, smoking, hyperlipidemia, obesity, migraine and the dependent variable lateral displacement of basilar artery, again only the presence of migraine was significantly associated with an altered lateral displacement of the basilar artery (*p* = 0.032).

The frequency distribution of the lateral displacement of basilar arteries in migraine patients showed a peak shift to the right (Fig. 5B and 5C). Taking the median lateral displacement of basilar arteries in migraine patients (3.9 mm) as a cutoff, subgroups could be identified with higher (*n* = 30, 48%) and normal (*n* = 33, 52%) lateral displacements. No differences in age, gender or risk factors were observed when these groups were compared.

When this cutoff value was applied, 22% of control subjects had a higher than median lateral displacement of the basilar arteries, which is significantly less than in the migraine group (chi-square test *p* = 0.000).

### Elongation

Forty-five of the 63 patients with migraine (71%) had a reduced SUCA outlet angle (< 160°) or a lateral displacement of the basilar artery (> 3.9 mm). Nineteen of the 63 patients with migraine (30%) had both basilar elongation features.

When only migraine patients with elongation features were analyzed, aura symptoms occurred more often in migraine patients with both elongation features than in patients with one elongation feature (chi-square test 0.037). There were no differences between episodic and chronic migraine patients (chi-square test 0.909).

Forty-one out of the 126 control subjects (33%) had a reduced SUCA outlet angle (< 160°) or a lateral displacement of the basilar artery (> 3.9 mm). Fourteen of the 126 control subjects (11%) had both basilar elongation features.

## Discussion

In this study, we found a lower SUCA outlet angle and a greater lateral displacement of the basilar artery in migraine patients compared to age- and gender-matched controls. Both findings suggest an elongation of the basilar artery as well as the intracranial vertebral arteries and their branches. This vertebrobasilar elongation affect mainly migraine patients with aura.

We have no firm evidence that the reported anatomic changes directly cause or promote migraine disease or attacks. Possible mechanisms are discussed below. These ideas must be handled with caution, as it is at least as likely that the findings reflect an epiphenomenon and not a direct chain of causation.

The smoker criteria were previously used to characterize basilar artery dilation and elongation [25]. They assess the diameter and lateral displacement of the basilar artery and the height of the basilar head. These criteria have limitations since external influences affect these characteristics; the diameters of the cerebral arteries have a complex relationship [32], and the basilar artery diameter is influenced by gender [23] and the volume of the supplied flow area. A bilateral aplasia of the precommunicating segment of the posterior cerebral artery, for example, results in a small basilar diameter. Determination of the angiographic diameter has a relevant inaccuracy [33]. The often detectable lateral displacement of the basilar artery in migraine patients with aura [23, 24] could be due to contralateral hypoplasia of a vertebral artery [34] since such vertebral hypoplasia is frequently seen in migraine patients with aura [35]. The height of the basilar head is mainly determined by the fusion of the basilar artery during embryonic development. All these influences complicate the acquisition of basilar abnormalities.

Due to the problems of detecting abnormalities in the basilar artery, the SUCA outlet angle could be an optimal way to characterize a cranial displacement of the distal basilar artery and thus determine the condition of the vertebrobasilar arteries [27]. We chose to examine the basilar artery because dilation and elongation are most likely to occur there [26]. A decrease in the SUCA outlet angle is most likely due to an elongation of the basilar artery as well as the intracranial vertebral arteries and their respective branches.

A decreased SUCA outlet angle has already been described in patients with adult Pompe disease, which has similar vascular complications to migraine (e.g., increased vascular stiffness and arterial dissections) [36, 37]. The decreased angle is a surrogate for a vertebrobasilar elongation that has been shown in many studies [38–41]. Histological findings have revealed that a fragmentation of the elastic fibers in the arterial walls may be a possible cause in adult Pompe patients [42].

Vertebrobasilar elongation in migraine patients is also supported by the increased lateral displacement of the basilar artery. Our findings are in line with previous data, as a lateral displacement of the basilar artery has already been identified in migraine patients with aura [23, 24].

Observing the stabilizing structures could determine whether elongation of the basilar artery results in lateral displacement or cranial displacement. For example, a bilaterally strong anterior inferior cerebellar artery could impede lateral displacement. Both lateral and cranial displacement were present in 30% of the migraine patients; this could be due to a distinctive weakness of the arterial walls or strains.

In migraine patients, various mechanisms can alter the vertebrobasilar wall structures, causing an elongation in the arteries. The underpinnings remain speculative, but there are some indications worth considering.

Genome-wide analyses have shown that a locus for the LDL receptor related protein 1 (LRP1) gene is most strongly associated with migraine disease [2, 3]. Among its other functions, it protects the arterial wall by reducing the effects of proteases such as elastase and metalloproteinase MMP-9 [4]. Elastase activity [43] and MMP-9 levels [44, 45] are elevated in migraine patients. Elastase leads to inflammation in the arterial wall with secondary fragmentation of the elastic fibers [46, 47]. This leads to elongation and increased vessel stiffness [48, 49]. The basilar artery is likely to be predominantly affected as it has a particularly high concentration of elastin [50]. Furthermore, MMP-9 leads to a degradation of collagen and other extracellular matrices, which also results in damage to the structural integrity of the arterial wall. Taken together, the increased activity of LRP1-regulated elastase and metalloproteinase MMP-9 leads to a weakening and elongation of the arterial wall. Such weakening and elongation explain the increased arterial stiffness, decreased cerebrovascular hypercapnia reactivity, complications associated with a weakening of the arterial wall (e.g., hemorrhagic strokes and aneurysmal formation), decreased SUCA outlet angle, and lateral displacement of the basilar arteries in migraine disease. Same accounts for dissections due to chiropractic manipulations or sudden head movements [51]. Cranial displacement of the vertebrobasilar arteries would also explain the clustered trigeminal neuralgia or oculomotor cranial nerve palsies in migraine [19, 20].

Other organs rich in elastin or collagen might also show changes in migraine patients. Furthermore, the distensibility of the internal jugular vein is reduced in migraine. Such venous outflow obstruction could lead to venous hypertension and congestion [8], which was previously shown to cause migraine like symptoms as headache, nausea, and vomiting also apart from migraine disease [52]. The aorta is rich in elastin and a cluster of abdominal aortic aneurysms has been reported [9]. The optic nerve has also been shown to have an increased stiffness in patients with migraine [53]. An ultrastructural examination of the skin and skeletal muscle arteries of migraine patients has revealed disturbances in the extracellular matrix and an elastinopathy with dark deposits in the elastin layer [54].

More specifically, a decrease in the SUCA outlet angle may, for example, result in a narrowing of the lumen at the SUCA outlet. Such stenosis could explain the increased incidence of border zone infarcts in the SUCA territory, as shown in the population-based CAMERA study [55]. However, the findings of this study should be regarded with caution because the individual border zones are very flexible and cannot be assessed in individual cases without the use of special techniques (e.g., synchronous ink injections).

In migraine patients, the repeated influence of headache medication or vasoactive drugs, e.g., triptans, must also be considered. An influence caused by triptans cannot be ruled out completely as the effect of vasoconstrictive agents on the elongation of the basilar artery has yet to be studied. Endothelial dysfunction [56, 57], increased blood flow and shear stress in the basilar artery [8, 58], increased oxidative stress [59–61], and inflammatory responses in the arterial walls [62] may also damage the walls of the vertebrobasilar arteries in migraine patients. It remains unclear whether an elongation of vertebrobasilar arteries with reduced SUCA outlet angles leads to relative cerebellar hypoperfusion, which then causes a release of CGRP. This increased level of CGRP may be directly linked to migraine attacks.

It is worth noting that only 71% of the examined migraine patients were affected by this vertebrobasilar elongation (SUCA outlet angle < median of migraine patients, or lateral displacement of basilar artery > median of migraine patients). Since migraine is a polygenic disorder and is diagnosed solely on the basis of clinical symptoms, there is a high probability that various -as of yet unknown -processes are hidden behind the diagnosis. The different behavior of the various subgroups is very consistent with the diversity of migraine, as the migraine attack is probably the terminal stage of various pathophysiological mechanisms and not all underlying mechanisms may affect the vertebrobasilar arteries.

The main limitations of this study are its retrospective design and the classification of migraine based on clinical documentation. It is unlikely that headache diaries were evaluated during inpatient stays. Thus, the differentiation between episodic/chronic migraine and migraine with/without aura may not be reliable**.** Furthermore, some migraine diagnoses may have been missed, as patients tend to underreport if not actively asked. This would lead to an underestimation of the reported effects on the elongation of the basilar artery. The use of former inpatients means that the collective studied is not representative. The proportion of migraine patients with aura but also of chronic migraine patients is higher than in the normal population. Migraine patients with pre-existing vascular conditions, e.g., aneurysm, stroke or dissection, were excluded from the study. A comparison with a clinical control group reflects the real situation in everyday hospital practice. In healthier controls with fewer cardiovascular risk factors (e.g., diabetes mellitus, hyperlipidemia), an even higher SUCA outlet angle or lower lateral displacement of the basilar artery may be assumed. Another weakness of this study is that we did not analyze anterior or posterior displacement of the basilar artery, but there is no reason to assume that changes in this plane would contradict our findings.

In summary, this study found that SUCA outlet angles and the lateral displacement of the basilar artery differed between migraine and control subjects. The SUCA angle represents a new and previously understudied parameter for describing the cranial displacement of vertebrobasilar arteries. The findings of this study should be verified prospectively. A much larger study format could enable the establishment of standard values for this measurement. The investigation of vascular alterations and complications in migraine patients is of great interest to researchers and could be supported by the easily feasible measurement of the SUCA outlet angle or lateral displacement of the basilar artery in the future.

This study provides an indication that intracranial, vertebrobasilar arteries are elongated in migraine patients. This elongation mainly affect migraine patients with aura.

## Data Availability

The datasets generated during and/or analyzed during the current study are available from the corresponding author on reasonable request.
